# Interpretation and Physical Meaning of Kinetic Parameters Obtained from Isoconversional Kinetic Analysis of Polymers

**DOI:** 10.3390/polym12061280

**Published:** 2020-06-03

**Authors:** Nicolas Sbirrazzuoli

**Affiliations:** Institut de Chimie de Nice, Université Côte d’Azur, CNRS, UMR 7272, 06108 Nice, France; Nicolas.SBIRRAZZUOLI@univ-cotedazur.fr

**Keywords:** isoconversional kinetic analysis, thermosetting polymers, semi-crystalline polymers, biobased polymers, nanocomposites

## Abstract

Several successful examples—where physically sounded kinetic information was obtained from thermoanalytical data in different application fields, such as polymerization of thermosetting resins, biobased polymers and nanocomposites, crystallization and glass transition of semi-crystalline polymers and their nanocomposites—are here presented and discussed. It is explained how the kinetic parameters obtained from advanced isoconversional methods can be interpreted in terms of reaction mechanisms or changes in the rate-limiting step of the overall process, in the case of complex chemical reactions or complex physical transitions, and how these parameters can be used to extract model-fitting parameters.

## 1. Introduction

Obtaining kinetic parameters with a real physical meaning in the case of complex reactions is not straightforward. Isoconversional methods are amongst the more reliable kinetic methods for the treatment of thermoanalytical data [1,2]. These methods are very effective when the reaction mechanism is not known and/or in the case of complex reactions involving several parallel, consecutive steps, or a combination of these, as well as large variations in viscosity. The main advantages of isoconversional methods are that they afford an evaluation of the effective activation energy, *E*_α_, without assuming any form of the reaction model, and can detect changes in the rate-limiting steps of the overall reaction rate as measured with thermoanalytical techniques. Changes in the reaction mechanism are obtained by analyzing the changes in the *E*_α_ variation, called the *E*_α_-dependency. For this reason, isoconversional methods have largely been used for studying the polymerization kinetics of numerous thermoset resins, but have also been found to be very efficient in the elucidation of physical transition kinetics, such as the crystallization and glass transition of polymers, the gelation of biopolymers, and melting [1,3]. This article aims at presenting several successful examples where physically sounded kinetic information was obtained from thermoanalytical data in different application fields, and explaining how the kinetic parameters obtained can be interpreted in terms of reaction mechanisms or changes in the rate-limiting step of the overall process. These examples show that advanced isoconversional methods provide for the obtaining of kinetic parameters with a real physical meaning and, in some cases, can be used to extract model-fitting parameters. These methods were initially applied with success to petro-based polymers, and are now frequently used in this field, thus the present study aims at promoting their use in the field of biobased and biodegradable polymer chemistry.

## 2. Theoretical Part

### 2.1. Advanced Isoconversional Methods

The isoconversional principle states that the reaction rate at a constant extent of conversion is only a function of temperature. Thus, it is possible in this case to compute a value of *E*_α_ for each value of α without any assumption regarding the reaction model.

The general form of the basic rate equation (d*α*/d*t*) is usually written as [1]:

(1) $$\frac{d\alpha }{dt}=k(T)f(\alpha )=A\exp\left(-\frac{E}{RT}\right)f(\alpha )$$

where *k*(*T*) is the rate constant, *T* is the temperature, *f*(*α*) is the differential form of the mathematical function that describe the reaction model that represents the reaction mechanism, *E* is the activation energy, and *A* is the pre-exponential factor. Linearization of Equation (1) leads to Equation (2) which, if computations are performed at a constant *α* value, is known as the Friedman’s method:

(2) $$\ln{\left(\frac{d\alpha }{dt}\right)}_{\alpha ,i}=\ln\left[A_{\alpha }f(\alpha )\right]-\frac{E_{\alpha }}{RT_{\alpha ,i}}$$

To use this Equation, one should determine the reaction rate (d*α*/d*t*)_α,*i*_ and the temperature *T*_α,*i*_ corresponding to a given extent of conversion *α* for the *i* temperature programs used. For a given extent of conversion, the term [*A_α_ f*(*α*)] is constant, and the plot on the left hand side of Equation (2) versus the reciprocal of temperature gives a straight line, which allows us to obtain a value of E for each value of *α*, named *E*_α_, without any assumption regarding the reaction model *f*(*α*). Note that using this method it is not possible to independently evaluate *A_α_* and *f*(*α*) without introducing the reaction model in the form of the function *f*(*α*). The advantages of differential methods, such as Friedman’s method, are that they use no approximations and can be applied to any temperature program, i.e., isothermal, nonisothermal, or even more complex temperature programs. In such a way, the sample temperature, if available, can be used for the kinetic computations. The main drawback is that differential isoconversional methods can sometimes reveal numerical instabilities [4]. To overcome some drawbacks of differential and usual integral methods, advanced isoconversional methods have been developed [1,2,3]. One of these methods is the advanced non-linear isoconversional method (NLN), or Vyazovkin’s method, described by Equations (3) and (4) [5,6,7].

(3) $$\Phi (E_{\alpha })=\sum_{i=1}^{n}\sum_{j\neq i}^{n}\frac{J\left[E_{\alpha },T_{i}(t_{\alpha })\right]}{J\left[E_{\alpha },T_{j}(t_{\alpha })\right]}$$

(4) $$J\left[E_{\alpha },T(t_{\alpha })\right]=\int_{t_{\alpha -\Delta \alpha }}^{t_{\alpha }}\exp\left[\frac{-E_{\alpha }}{RT(t)}\right]dt$$

where *E*_α_ is called the effective activation energy. The *E*_α_ value is determined as the value that minimizes the function Φ(*E*_α_). A value of *E*_α_ is computed for each value of *α* lying in between 0.02 and 0.98 with a step of 0.02. For each *i*-th temperature program, the time *t*_α,i_ and temperature *T*_α,i_ related to selected values of α are determined by an accurate interpolation, using a Lagrangian algorithm. This nonlinear kinetic method (NLN) allows one to handle a set of n experiments carried out under different arbitrary temperature programs *T*_i_(*t*), and uses a numerical integration performed using the trapezoidal rule of the integral with respect to time. The method developed by N. Sbirrazzuoli and implemented in his software can treat any kind of isothermal or non-isothermal data from differential thermal analyses (DTA), differential scanning calorimetry (DSC), calorimetry (Calvet-type and reaction calorimetry), fast scanning calorimetry (FSC), thermogravimetric analysis (TGA), dynamic mechanical analysis (DMA), temperature modulated DSC (TMDSC) rheometry, heat capacity variations, or other techniques [4,6,8,9,10].

Application of Equations (1)–(4) requires evaluation of the extent of conversion at time *t*, *α*_t_. This parameter can be extracted from data provided by different techniques by assuming that the measured property is proportional to the reaction progress. If one uses DSC or calorimetric data, the extent of conversion is computed according to Equation (5)

(5) $$\alpha_{t}=\frac{\int_{t_{i}}^{t}{\left(dQ/dt\right)}_{}dt}{\int_{{}_{t_{i}}}^{{}_{t_{f}}}{\left(dQ/dt\right)}_{}dt}=\frac{Q_{t}}{Q_{tot}}$$

where *t*_i_ represents the time at first integration bound of the DSC signal, and *t*_f_ the time at last integration bound selected when the reaction is finished. d*Q*/d*t* is the heat flow measured by DSC at time *t*, *Q*_tot_ is the total heat released or absorbed by the reaction and *Q*_t_ is the current heat change.

For integral data, such as TGA, DMA, rheometry or heat capacity data, the extent of conversion is computed according to Equation (6)

(6) $$\alpha_{t}=\frac{X_{t}-X_{t_{i}}}{X_{t_{f}}-X_{t_{i}}}$$

where *X* represents any physical property measured at time *t*.

### 2.2. Interpretation of the E_α_-Dependency

It has taken time for model-free methods, mainly represented by isoconversional methods, to be accepted by the kinetic community. Indeed, the proposed approach constitutes a real paradigm-change. The classical kinetic approach starts by writing the supposed mechanism of the reaction and then the kinetic parameters are deduced, most often by fitting the data. In the case of complex reactions, an accurate fit of the data can be obtained with kinetic parameters without physical meaning. The model-free approach is completely different, since it involves analyzing the kinetic data obtained without formulating hypotheses on the reaction mechanisms, and trying to deduce information about a possible mechanism or a change in the rate-limiting step of the overall reaction rate that may occur during the reaction progress. This is accomplished by analyzing the so-called *E*_α_-dependency. In the case of a single-step process, *E*_α_ does not change with α. In this case, evaluation of kinetic parameters is quite easy. However, most reactions, and especially those occurring with high viscosity variations, do not obey this simple kinetic scheme. Significant variations in *E*_α_ with α indicate a kinetically complex process (i.e., multi-steps) and *E*_α_ variations can be associated with a change in the reaction mechanism. Recently, Sbirrazzuoli has refined this approach by pointing out the interest in using variations of *A*_α_, *f*(*α*) and *k*(*T*), evaluated in a model-free way, to identify rate-limiting steps [11].

The way to evaluate independently *A*_α_ and *f*(*α*) in a model-free way is explained in detail in [10,11]. The study highlighted correlations between the reaction rate, activation energy dependency, rate constants for the chemically controlled part of the reaction and the diffusion-controlled part, activation energy and pre-exponential factors of the individual steps, and changes in the rate-limiting steps. It was shown how some parameters computed using Friedman’s and NLN methods can help to identify change in the rate-limiting steps of the overall polymerization mechanism as measured by thermoanalytical techniques. The principle is based on the idea that the overall reaction rate (d*α* /d*t*)_α_, calculated for a given extent of conversion (*α*), can be put in the form of a product of two terms, [*A_α_ f*(*α*) and exp(−*E*_α_/R*T*)]. If the rate decreases and one of the two terms increases while the other decreases, it means that this term has become dominant, which indicates a change in the limiting step of the reaction. In addition, calculating *A*_α_, *f*(*α*) and *k*_α_(*T*) separately (but in a “model-free” way) using the method of compensation effect allows one to distinguish a change in the rate due to a change in the number of effective collisions or “entropic” effects (i.e., term *A*_α_), or due to a change in the reaction mechanism (i.e., term *f*(*α*)) [10].

## 3. Discussion

### 3.1. Polymerization and Crosslinking

As for any chemical reaction, the overall reaction rate of polymerization and crosslinking reactions depends on several factors—such as reactivity of chemical species, activities of reactants, temperature, number and efficient collisions between molecules, and the energetic barrier to overcome for reactants to be transformed into products—but diffusion becomes the rate-limiting factor when molecular motion becomes hindered by the growing polymer chains. After the gel point, the mobility is hindered in the polymer network, and molecules can only participate in a reaction after a series of diffusion jumps. Crosslinking of the epoxy–amine system has demonstrated a decrease of *E*_α_, from high values to very low values [12]. The initial high values correspond to the activation energy of the uncatalyzed reaction (70–90 kJ mol·^1^), while the energy of the catalyzed reaction corresponds to lower values (45–55 kJ mol·^1^) [12,13]. In these pioneering works, the two systems under study were respectively an epoxy novolac (Ciba-Geigy; araldite LY 5052) cured with isophorone diamine, and diglycidyl ether of bisphenol A (DGEBA) cured with 1,3-phenylenediamine. Although these systems were not biobased, it will be shown in the following that the methodology developed several years ago can be applied to the development of biobased polymers. In addition, as the initial methods have been refined since their first applications, more information on mechanisms can be obtained nowdays. The very low values reported at the end of the reaction (10–20 kJ mol·^1^) are related to the diffusion of small monomers. The overall rate can be parametrized in term of an autocatalytic process during the chemically controlled part of the reaction, followed by a diffusion model for the late stages of the reaction. This result has been confirmed by numerical simulations [11].

#### 3.1.1. Sourour and Kamal Model

The initial stages of the reaction can be described by the autocatalytic model proposed by Sourour and Kamal, where the rate constant *k*(*T*) of Equation (1) is expressed as Equation (7) [14].

(7) $$k_{C}\left(T,\alpha \right)=A_{1}\exp\left(-\frac{E_{1}}{RT}\right)+A_{2}\exp\left(-\frac{E_{2}}{RT}\right)\alpha^{m}$$

*A*_1_ being the pre-exponential factor for the non-catalyzed reaction and *A*_2_ the pre-exponential factor for the catalyzed reaction. *E*_1_ and *E*_2_ respectively represent the activation energy of the non-catalyzed reaction and the activation energy of the catalyzed reaction, and *m* and *n* are kinetic exponents.

#### 3.1.2. Diffusion Model

The Sourour and Kamal model is not suitable to describe the end of the polymerization process when the reaction is diffusion-controlled, thus a rate constant that takes into account the diffusion processes has been proposed [12,15];

(8) $$k_{D}\left(T,\alpha \right)=D_{0}\exp\left(-\frac{E_{D}}{RT}+K\alpha \right)$$

where *D*_0_ represents the pre-exponential factor of the diffusion-controlled reaction, *K* is a constant accounting for the effect of the chemical reaction on the change in diffusivity, and *E*_D_ represents the activation energy of the diffusion-controlled reaction.

#### 3.1.3. Effective Activation Energy

Application of the isoconversional principle to the autocatalytic model of Sourour and Kamal leads to Equations (9)–(11) [11,12]:

(9) $$E_{\alpha }=\frac{k_{1}\left(T\right)E_{1}+k_{2}\left(T\right)E_{2}\alpha^{m}}{k_{1}\left(T\right)+k_{2}\left(T\right)\alpha^{m}}$$

(10) $$E_{\alpha }=\frac{A_{1}\exp\left(-E_{1}/RT\right)E_{1}+A_{2}\exp\left(-E_{2}/RT\right)E_{2}\alpha^{m}}{A_{1}\exp\left(-E_{1}/RT\right)+A_{2}\exp\left(-E_{2}/RT\right)\alpha^{m}}$$

(11) $$E_{\alpha }=\frac{\left(A_{1}/A_{2}\right)\exp\left(-E_{1}/RT\right)E_{1}+\exp\left(-E_{2}/RT\right)E_{2}\alpha^{m}}{\left(A_{1}/A_{2}\right)\exp\left(-E_{1}/RT\right)+\exp\left(-E_{2}/RT\right)\alpha^{m}}$$

These equations show that the effective activation energy depends on both temperature and the extent of conversion. The activation energies of the non-catalyzed (*E*_1_) and catalyzed (*E*_2_) reactions are obtained by non-linear fitting of Equation (11) to the effective activation energy dependency with temperature. The fit is realized in the interval where the reaction is chemically controlled (generally for *α* around 0.3–0.5), and leads to the activation energies *E*_1_ and *E*_2_ [11,12]. Note that at a low degree of conversion (*α* → 0), the effective activation energy *E*_α_ yields an estimate for the activation energy of the non-catalyzed reaction *E*_1_.

Application of the isoconversional principle to the diffusion model leads to Equations (12)–(14) [11,12]:

(12) $$E_{\alpha }=\frac{k\left(T\right)E_{D}+k_{D}\left(T,\alpha \right)E_{2}}{k\left(T\right)+k_{D}\left(T,\alpha \right)}$$

(13) $$E_{\alpha }=\frac{A_{2}\exp\left(-E_{2}/RT\right)E_{D}+D_{0}\exp\left(-E_{D}/RT+K\alpha \right)E_{2}}{A_{2}\exp\left(-E_{2}/RT\right)+D_{0}\exp\left(-E_{D}/RT+K\alpha \right)}$$

(14) $$E_{\alpha }=\frac{\left(A_{2}/D_{0}\right)\exp\left(-E_{2}/RT\right)E_{D}+\exp\left(-E_{D}/RT+K\alpha \right)E_{2}}{\left(A_{2}/D_{0}\right)\exp\left(-E_{2}/RT\right)+\exp\left(-E_{D}/RT+K\alpha \right)}$$

A non-linear fitting of Equation (14) is realized in the interval where the reaction is diffusion-controlled, and leads to the activation energies *E*_2_ and *E*_D_.

**Figure 1:**
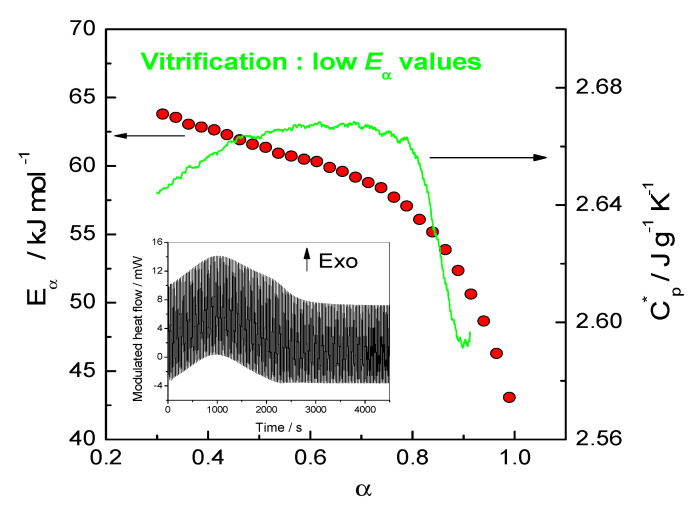
Effective activation energy (*E_α_*) (left) and complex heat capacity (*C*_p_*) obtained from quasi-isothermal TMDSC measurements (right) vs extent conversion (*α*) during isothermal curing. Inset: Total TMDSC heat flow vs time. Adapted with permission from Copyright^©^ 2002 Elsevier Ltd. All rights reserved [18].

Estimation of the activation energy of multi-step processes can be simplified by using a large excess of one reactant. As an example, a large excess of amine (1,3-phenylenediamine) was used to estimate the activation energy of primary amine addition (non-catalyzed) independently of that of the secondary amine addition to epoxy. This was possible because the reactivity of the former is much higher than that of the latter. Indeed, the use of a large excess of amine has led to a quasi-constant value of the activation energy, which attests to the control of the overall rate by a single-step process in this case [16]. If *k*_1_(*T*) ≫ *k*_2_(*T*) then *E*_α_ → *E*_1_ in Equation (9). The obtained value was assigned to the values of the activation energy of the primary amine addition (55 kJ mol·^1^). For the stoichiometric counterpart of this system, it was shown that the reaction shift from chemical to diffusion control for *α* > 0.30. This was first deduced from the sole *E*_α_–dependence analysis, obtained from DSC measurements and confirmed later by TMDSC and rheological measurements [12,16]. Figure 1 illustrates the correlation between the decrease of *E*_α_ and of the complex heat capacity (*C*_p_*) attributed to vitrification. Vitrification during isothermal crosslinking can be detected by rheometry or TMDSC measurements [16,17]. After this stage, the molecular mobility is considerably reduced, and the reaction is diffusion controlled.

**Figure 2:**
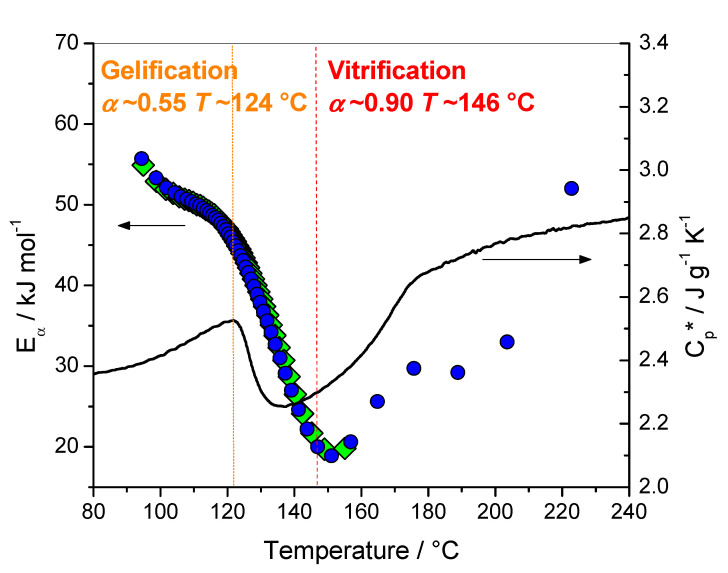
Effective activation energy (*E_α_*) vs temperature during nonisothermal curing at 1, 2 and 4 °C min·^1^ (left) and complex heat capacity (*C*_p_*) obtained from nonisothermal TMDSC measurement at 1 °C min·^1^ (right). The dot lines indicate the temperature at gelation and vitrification measured by oscillatory rheometry; the subsequent extent of conversions was obtained using DSC and rheometry. Lozanges: last integration bound between 149 and 205 °C; circles: last integration bound between 228 and 297 °C. Adapted with permission from Copyright^© 2020^, John Wiley and Sons and adapted with permission from Copyright^©^ 2003 Elsevier Ltd. All rights reserved [16,20].

In the nonisothermal mode, the *E*_α_ values obtained at the end of the reaction are very low (10–20 kJ mol·^1^), and do not match with the activation energy of any chemical reaction, such as primary and secondary amine addition or etherification (Figure 2). In fact, these values correspond to the diffusion of small molecules, such as the unreacted chain segments of the polymer chain [19]. At the gel point the growing individual macromolecules become connected into a single network so that the epoxy system loses the ability to flow, however, it is still capable of a long-range motion of polymer chain segments if the temperature is increased, as in nonisothermal conditions. This results, in this case, in the increasing *E*_α_-dependencies at the end of the reaction, instead of the decreasing tendency observed for reactions controlled by motions of small molecules [20,21]. This is explained by the devitrification and/or reactivation of the chemical reaction due to an increase in molecular mobility. Devitrification can be detected by the increase of the complex heat capacity *C*_p_* measured by TMDSC. An example is reported on Figure 2, where devitrification occurs for a temperature higher than 140 °C. It is worth noting that this temperature domain is in perfect agreement with the increase of *E*_α_ attributed to the reactivation of chemical reactions (Figure 2). The agreement between *E*_α_-dependencies and *C*_p_* variations presented in Figure 1 and Figure 2 is remarkable, and confirms that the *E*_α_-dependence reflects a change in the overall polymerization mechanism.

Combining DSC and oscillatory rheometry, it was reported for this system that gelation occurs at *α* around 0.55 at a temperature of 124 °C, and vitrification at *α* around 0.90 at a temperature of 146 °C [16]. These values are in perfect agreement with the data of Figure 2. The temperature of 124 °C corresponds to the beginning of the *C*_p_*’s decrease, while the temperature of 146 °C corresponds to the minimum of *E_α_* (note that for nonisothermal conditions the heating rates used for the evaluation of *E*_α_ are higher than the rate used for the measurement of *C*_p_*, which induces a slight shift of the *E*_α_ values to a higher temperature). For a temperature higher than 140–150 °C devitrification occurs, as attested by the *C*_p_* increase, and chemical reactions are reactivated.

The control via diffusion of monomers at the end of the reaction is considerably reduced when nanoclays are added to the epoxy–amine system (DGEBA/1,3-phenylenediamine) [22]. The molecular mobility is higher at the end of the reaction for mixtures containing clay independent of the nature of the alkylammonium ions present in the galleries of the clay, as reflected by the lower decrease of *E_α_*, characteristic of diffusion control. Using the method proposed by Sbirrazzuoli, based on Equations (11) and (14), activation energies consistent for the uncatalyzed and for the catalyzed reaction were obtained [13]. In addition, it was shown that addition of modified clay induces less ineffective shocks, and the reactivity is increased due to an enhancement of the efficiency of collisions [13]. For the chemically controlled part of the reaction, the high increase in the efficiency of collisions of the amine addition, initiated by a proton donor, results in an enhancement of the ring-opening. In the diffusion-controlled part of the reaction, the pre-exponential factor, computed according to the model-free method described, is also increased by addition of modified clay, and was explained by a high increase of the frequency of diffusion jumps (1000 times) [10,13]. A similar result was reported for Furfuryl alcohol (FA) polymerization in presence of organically modified montmorillonite [23].

Because the fit of the *E_α_*-dependence alone may lead to several sets of correlated pre-exponential factors (*A*_1_, *A*_2_, *D*_0_), it is preferable to use first the *E_α_*-dependence to get initial values and to use the ratios (*A*_1_/*A*_2_, *A*_2_/*D*_0_) as constraints [13]. Thus, Equations (11) and (14) were proposed by Sbirrazzuoli [13] to reduce the degree of freedom and avoid obtaining local minima when applying non-linear minimization. Only ratios are obtained. Then, the values *A*_1_, *A*_2_, *D*_0_ are obtained by fitting experimental rates and Equations (10) and (13) as additional constraints [24]. The use of the reaction rate at a constant value of the extent of conversion [d*α*/d*t*]_α*,i*_ (see Equation (6) of reference [11]) facilitates the computations because a lower number of points is involved in the minimization [11]. These data ([d*α*/d*t*]_α*,i*_) are automatically computed when one use the Friedman method. 

For the chemically controlled part of the reaction studied, an apparent contradiction was reported [13]. The reaction with nanoparticles started at a lower temperature, while the values of *E_α_* were higher. This was explained by the higher values found for the pre-exponential factor when nanoparticles were introduced. Indeed, a higher value of *A* resulted in an increase of the reaction rate, which compensated for the increase of *E* (Equation (1)). This experimental observation was confirmed later using simulated data [11]. These simulations have confirmed the relevance of the use of isoconversional analysis to identify the rate-limiting steps in complex (multi-step) reactions. These examples illustrate the significance of computing the pre-exponential factor dependence (ln*A_α_*-dependence) in a model-free way using compensation effect, in addition to *E_α_*. Then, once *E_α_* and *A_α_* dependencies have been determined, the effective rate constant (*k*_ef_) and mathematical function related to the mechanism *f*(*α*) can be evaluated for each extent of conversion. These parameters are then used to identify changes in rate-limiting steps and to gain insights into the reaction mechanism. 

Mechanistic insights were obtained for the polymerization of cyanate esters with different bridging fragments ((CH_3_)_2_C, S, and O) using isoconversional kinetic analysis. A higher reactivity of a sulfur-bridged monomer compared to its carbon-bridged analog was found. This was explained by the larger value of the pre-exponential factor for the metal-catalyzed step of the reaction, due to a higher polarizability that induces a stronger intermolecular interaction between molecules and promotes preferred orientation of the reacting monomers [24].

Granando et al. reported a quasi-constant value of *E_α_* for the crosslinking of a Phenol–Formaldehyde (PF) resole system, which was attributed to a one-step mechanism of condensation of the methylol group [25]. They concluded that the diffusion-controlled regime is much less limiting than in epoxy systems, where the curing kinetics drastically slows down. This system was compared to the crosslinking of a phenol–terephthalaldehyde (PTPA) resole, which exhibits a more complex mechanism. The first plateau of *E_α_* was assigned to the condensation of the secondary alcohol onto phenols, followed by a slight decrease associated with the shift in the polymerization mechanism, from the first reaction to the addition onto phenol of the second aldehyde function for the one-fold reacted TPA. The slight increase of *E_α_* during the later stage of curing PF and PTFA was assigned to diffusional restrictions due to the forming of a three-dimensional crosslinked network. 

**Figure 3:**
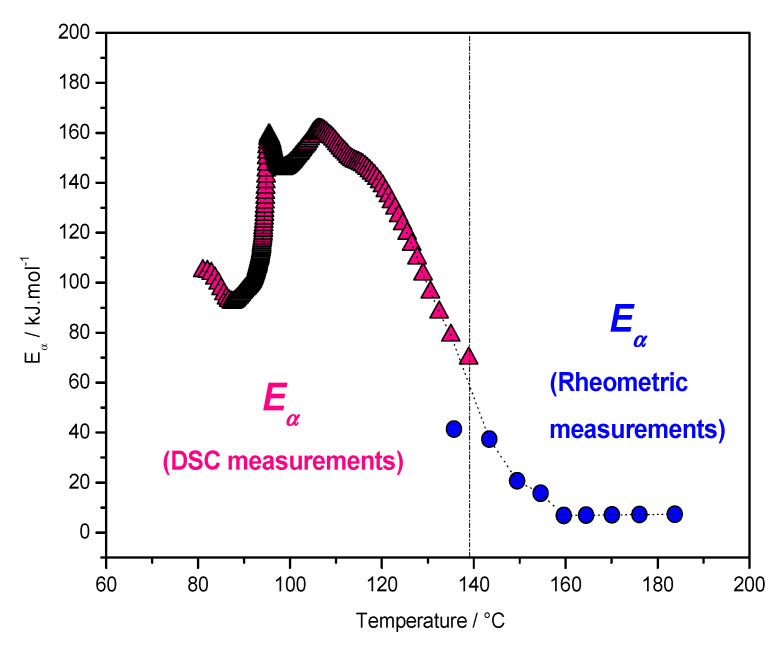
Effective activation energy dependency (*E_α_*) as a function of temperature for FA/I30E system, by DSC (triangle) and by rheometry (circle) [26].

The polymerization kinetics of Furfuryl alcohol (FA) into PolyFurfuryl alcohol (PFA) with organically modified Montmorillonite (Nanomer I30E) under acidic catalysis were studied by both DSC and rheometry [26]. DSC can detect residual heat after curing, but it is generally not easy to use these data to compute kinetic parameters, especially at high degrees of crosslinking. In this case, rheometry can be an efficient technique following the end of the reactions occurring in a highly viscous media. Polymerization of FA into PFA generates water, which makes the rheological measurements difficult. Thus, for low temperatures DSC is favored, and at high temperatures rheology is more suitable when the objective is to use these data to make complex computations which require high-quality data. The perfect continuity between the *E_α_* values obtained from DSC and the rheometric data presented in Figure 3 is additional proof of the physical meaning of the *E_α_* values obtained. Indeed, the *E_α_*-dependence cannot be determined by DSC at a high temperature because no signal is detected at the end of the reaction when the material is highly crosslinked, however, the remaining crosslinking of the system under heating can be adequately determined by rheometric measurements and the *E_α_*-dependence computed using this technique. In this case, the parameter *X* of Equation (6), used to follow the residual reaction occurring in the rubbery state, is the elastic modulus *G*’.

**Figure 4:**
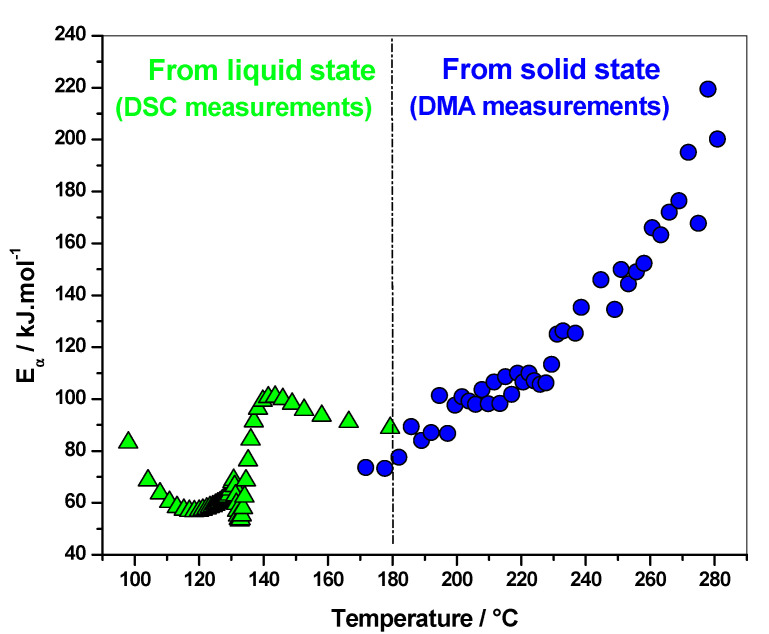
Effective activation energy dependency (*E_α_*) as a function of temperature obtained from DSC (triangle) and DMA (circle) measurements for FA/MA system [27].

Polymerization of FA into PFA with maleic anhydride (MA) as acidic initiator was studied by DSC and DMA [27]. DSC was used to monitor the reaction in the liquid state, while DMA was performed on cured materials, via heating samples from 20 to 350 °C following the residual reactions occurring in the solid state. An increase of the elastic modulus was observed for all the systems between 170 and 290 °C, while no thermal event was recorded at a temperature higher than 220 °C on the first DSC heating curves. Thus, this increase was attributed to residual crosslinks occurring in the rubbery state after intensive post-curing. The effective activation energy dependency (*E_α_*), as a function of temperature computed from DMA measurements, shows increasing values, which corresponds to a kinetic control by the diffusion of long polymeric chains (Figure 4). As the PFA material progressively cross-links in the solid state, the molecular mobility becomes more restricted, which leads to an increase in the activation energy. The values are lower in the presence of solvents, attesting that chains are less constraining in this case. More remarkably, there is a perfect continuity between the *E_α_*-dependencies obtained for polymerization from the liquid state (DSC measurements) and those obtained for additional cross-links occurring in the rubbery state (DMA measurements), the final values of the reaction starting from the liquid state being in good agreement with the first values of the reaction starting from the rubbery state. The combination of DSC and DMA data allows one to study the polymerization kinetics and get mechanistic information over a wide temperature range, starting from the liquid state and ending in the solid state. This perfect continuity is an additional indication of the physical meaning of the kinetic parameters obtained (Figure 4).

An increase of the *E_α_*-dependence attributed to kinetic control via the diffusion of long polymeric chains was also reported for the crosslinking of DGEBA with diamine, and for the polymerization of 2-hydroxyethyl methacrylate [20,21]. As already mentioned, the increasing dependence is expected when the process becomes determined by the diffusion of large molecules, such as polymer chains or their long segments. The difference between decreasing to very low values (5 kJ mol^−1^) obtained for the crosslinking of the FA/I30E system (Figure 3) and the increasing to high values (220 kJ mol^−1^) obtained for the FA/MA system (already cured) (Figure 4) also illustrate this statement [23,26,27]. In the case of the FA/I30E system, the elastic modulus reaches a plateau and the system is in the glassy state [23,26], while for the FA/MA system the elastic modulus starts to increase at high temperatures, when chemical reactions are re-activated. The *E_α_*-dependence decreases when the rate becomes limited by the diffusion of small molecules or short segments of the polymer chain, while the *E_α_*-dependence increase when the rate is limited by the mobility of long segments of the polymer chains [3]. Stochastically modulated temperature DSC has highlighted the vitrification and devitrification that may occur during crosslinking [16,28]. Vitrification induces a shift from chemical to diffusion control, which results in decreasing *E_α_*-dependency. In nonisothermal conditions, the system may devitrify after vitrification, leading to the reactivation of residual chemical reactions, which leads to increasing *E_α_*-dependency (Figure 2) [20].

Similar conclusions were drawn by analyzing the *E_α_*-dependencies resulting from the polymerization of epoxidized linseed oil (ELO) with three different biobased dicarboxylic acids, i.e., sebacic acid, suberic acid and succinic acid, used as crosslinkers [29]. The viscosity measured by nonisothermal dynamic rheometry of ELO cured with suberic or sebacic acids show a plateau at the end of the polymerization, while this was not the case when using succinic acid. In this case, the viscosity curve is steadily increasing, and the *E_α_*-dependency shows an increase at the end of the reaction. In contrast, when ELO is cured with suberic or sebacic acids, the *E_α_*-dependencies decrease to low values, as a result of the shift to diffusion control of small unreacted molecules, and chemical reactions are not reactivated at high temperatures in this case. In this study, the determination of *E*_α_, ln*A*_α_ and *f*(*α*) was done without assumption in the reaction mechanism. Indeed, evaluation of the kinetic triplet in a fully model-free way may allow new insights into the reaction mechanism to be obtained. This was achieved by coupling kinetic analysis, FTIR and chemo-rheology investigations. The consistency of the conclusions drawn from these different techniques for analyzing the complex polymerization mechanism is remarkable. It was shown that the early stage of the reaction is controlled by an autocatalytic step, which results from an increase of the amount of hydroxyl groups in the reaction medium for the three systems under studies, i.e., ELO/Succinic, ELO/Suberic and ELO/Sebacic. The formation of a sufficient amount of *β*-hydroxy-esters plays the major role while at low viscosity. For ELO/Suberic and ELO/Sebacic, the transport of unreacted molecules and/or their concentration in the rubbery state determine and explain the deceleration of the reaction rate at the end of the reaction. With succinic acid, the reaction starts at a lower temperature and the reaction rate is higher for the early stages of the reaction, while the apparent activation energy is higher. Again, this seems to be contradictory. Computations have shown that this phenomenon is due to a higher frequency of diffusion jumps, expressed by higher values of the pre-exponential term. For ELO/Succinic acid system, the viscosity is higher at the end of the process, and only an increase of the temperature permits an increase of the diffusion. At the end of the process, the viscosity is high and the number and efficiency of collisions between molecules are weak, and only an increase of the temperature permits an increase of the diffusion. The pre-exponential factor is much lower for longer chains (i.e., sebacic and suberic), leading to a much more pronounced difference in the *k*(*T*) values between ELO/Succinic and the two other systems. Such a difference, between the efficiency of collision for long chains and short chains, is somehow compensated by the influence of the reaction mechanism *f*(*α*). FTIR spectrum of the ELO/Succinic system did not highlight remaining carboxylic acid after cross-linking. This agrees with the lower *f*(*α*) values for this system, which attests to a progressively lower concentration of carboxylic group at the end of the reaction. The high decrease of the term *f*(*α*) explains the lower values of the reaction rate for succinic acid. The viscosity increase after the vitrification plateau for ELO/Succinic suggests the continuation of additional cross-links. As the temperature is continuously increasing, some chemical reactions can occur. This phenomenon involves a cooperative motion of long polymer chains formed during the crosslinking, and the energy barrier is higher, as compared to the low values obtained for the diffusion of small unreacted molecules in the case of sebacic and suberic acids. In contrast, the viscosity reaches a plateau for the ELO/Suberic and ELO/Sebacic systems at the end of the reaction where remaining carboxylic acids are still present, as shown by FTIR measurements. Nevertheless, these species are unable to react due to the poor transport of these longer chains, in agreement with a diffusion control evidenced by the decrease of *E*_α_ and *A*_α_ to low values at the end of the reaction. This is in perfect agreement with the shift from chemical to diffusion control of small molecules for curing with suberic and sebacic acids, and the diffusion control of long chain segment motions for curing with succinic acid. 

**Figure 5:**
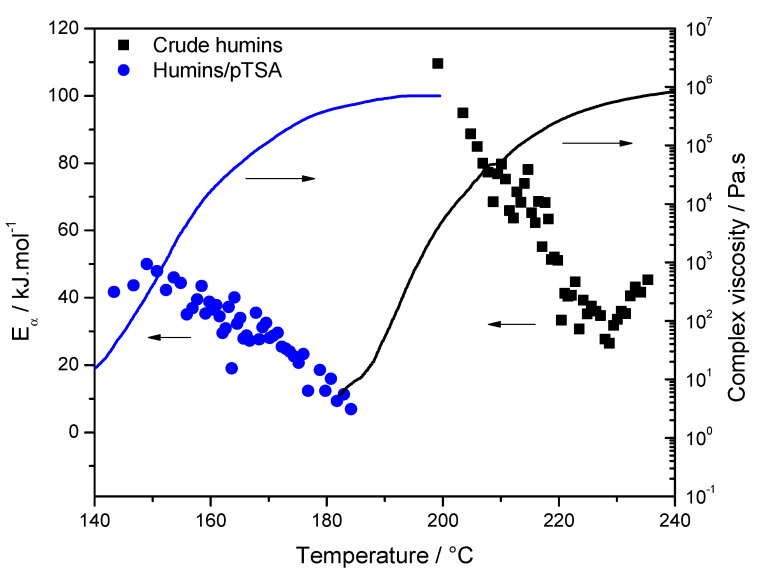
Left: evolution of the effective activation energy (*E*_α_) as function of the temperature for crude humins (black squares) and humins/*p*TSA (blue circles); and right: complex viscosity variation with temperature [30].

Humins are a biomass-derived material, a co-product of the acid-catalyzed conversion of cellulose and hemicellulose to platform chemicals. The *E*_α_ values computed from non-isothermal rheometric data on humins, and humins with acid initiator (*p*-Toluenesulfonic acid monohydrate; *p*TSA), show decreasing dependencies (Figure 5). The first steep decrease was attributed to an autocatalytic step. The first value of 110 kJ mol·^1^ represents the activation energy for the non-catalyzed crosslinking of crude humins. A second step is identified from *α* > 0.35, and at this stage the viscosity has strongly increased. The decrease to 25 kJ.mol·^1^ is in good agreement with a diffusion control at the end of the reaction. For *α* > 0.8, the high temperature reached might increase molecular mobility, promoting chemical reactions to be reactivated, and the rate is controlled by motions of longer chain segments, thus *E_α_* increases [30]. For humins with the *p*TSA system, the initiation reactions occur at significantly lower temperatures and with lower value of *E*_α_, which is in good agreement with the accelerating effect of *p*TSA.

In addition, the resulting *E_α_* decrease is in good agreement with the data obtained from isothermal DSC and complex heat capacity (*C*_p_*), obtained from quasi-isothermal Stochastically Temperature Modulated DSC (TOPEM^©^) [30].

### 3.2. Crystallization of Semi-Crystalline Polymers

At temperatures close to the melting temperature, the nucleation rate is low, and becomes the rate-limiting step for the crystallization rate of polymers from the melt. If the crystallization temperature is decreased, the crystal growth rate increases. This is typical of an anti-arrhenian behavior, which is explained by the control of the crystal growth rate by nucleation [3]. The opposite situation occurs when a sample is crystallized upon heating from the solid or glassy state. At a temperature above the glass transition temperature (*T*_g_) and well below the melting temperature (*T*_m_), the nucleation rate is high, and the diffusion is the rate-limiting step for the crystal growth rate. In this temperature domain, the crystallization rate obeys the classical arrhenius behavior. Application of isoconversional analysis to the non-isothermal crystallization of semicristalline polymers resulted in decreasing *E*_α_ values for crystallization upon heating from the solid state, and negative increasing *E*_α_ values for crystallization upon cooling from the melt [31,32,33]. It was shown that this result is in perfect agreement with the Hoffman–Lauritzen (HL) theory of secondary crystallization, which describes the dependence of the crystal growth rate on temperature. A consequence of this is that the effective activation energy *E*_α_, computed with an isoconversional method, should be negative and increasing in the melt crystallization region, and positive and decreasing in the glass crystallization region. A value of *E*_α_ = 0 should be theoretically obtained at the temperature of maximum growth rate, where the crystal growth rate reaches the maximum and the effective activation energy *E*_α_ changes from a negative to a positive value. 

Then, an entirely novel approach was proposed that allows for the evaluating of the parameters of the Hoffman–Lauritzen theory of polymer crystallization as derived via isoconversional analysis. This new method represents a unique way to extract important parameters regarding crystallization as well as additional information on the mechanisms, such as the anti-arrhenian dependence of the crystallization rate, with respect to the temperature and the mechanisms of chain folding. The dependence obtained gives real information regarding the changes in the mechanism during crystallization.

The Hoffman–Lauritzen theory describes the temperature dependence of the growth rate *G* measured microscopically, and is presented in Equation (15):

(15) $$G=G_{0}\exp\left(\frac{-U^{*}}{R(T-T_{\infty })}\right)\exp\left(\frac{-K_{g}}{T\Delta Tf}\right)$$

where *G_0_* is the pre-exponential factor, U^*^ is the activation energy of the segmental jump (associated with diffusion process), $T_{m}^{0}$ is the equilibrium melting temperature, ∆*T* = $T_{m}^{0}$ − *T* is the undercooling, *f* = 2*T*/($T_{m}^{0}$ + *T*) is the correction factor, and $T_{\infty }$ is a hypothetical temperature where motion associated with viscous flow ceases, which is taken 30 K below the glass transition temperature *T*_g_ (i.e., $T_{\infty }$ = *T_g_* − 30 K).

Application of the isoconversional principle results in:

(16) $$E_{\alpha }(T)=U^{*}\frac{T^{2}}{{\left(T-T_{\infty }\right)}^{2}}+K_{g}R\frac{{\left(T_{m}^{0}\right)}^{2}-T^{2}-T_{m}^{0}T}{{\left(T_{m}^{0}-T\right)}^{2}T}$$

where U* corresponds to the activation energy of molecular diffusion at the interfacial boundary between melt and crystals, and *K*_g_ corresponds to the activation energy for the nucleation of a crystal with critical size. According to the Hoffman–Lauritzen theory, the crystallization rate reaches the maximum for a given temperature, denoted by *T*_max_. If the crystallization temperature *T*_c_ is in the region *T*_max_–*T*_m_ (*T*_m_: melting temperature), the sample will follow an anti-Arrhenian behavior, which is characterized by the negative value of the temperature coefficient (represented by the apparent activation energy in our calculations), which increases when the temperature decreases (crystallization on cooling). In this region, the crystallization rate is controlled by nucleation, which is characterized by a negative temperature coefficient. If, on the other hand, the crystallization temperature *T*_c_ is in the region *T*_g_–*T*_max_, the sample will follow the classical Arrhenian behavior, which will result in positive values for the apparent activation energy as calculated with advanced isoconversional methods. In this temperature domain, the growth rate becomes controlled by diffusion and leads to a positive temperature coefficient, which decreases as the temperature increases because diffusion is facilitated (crystallization on heating). This new approach was applied with success to many different thermoplastic polymers [34,35,36,37,38,39,40,41,42,43,44].

As an additional proof of the physical meaning of this approach, in all these examples the overall shape of the *E_α_*-dependence computed with various isoconversional methods is always the same. Changes in the slope of this dependence were used to identify a change in the crystallization regime, while deviation from the variations predicted by the Hoffman–Lauritzen theory have allowed us to identify more complex phenomena not taken into account in this theory, such as primary nucleation, chain folding, lamellar thickening, etc. [38]. In contrast, the Avrami model is limited to a single-step process, which is generally not the case for polymers, as evidenced by the strong *E*_α_-dependencies frequently reported [31,32,33,35,36,37,38,39,40,41,42,43,44]. The physical meaning of the Avrami exponent remains questionable in the case of crystallization of polymers [38]. It was shown that application of the isoconversional principle to the Avrami equation leads to a constant value of *E*_α_, in contradiction with experimental observation [45]. An abrupt increase in the effective activation energy was observed for poly(ethylene therephtalate) (PET) at *α* ~ 0.85 (around 202 °C), and was assigned to a change in the crystallization mechanism, in agreement with microscopic measurements [31,32]. This result shows that the negative values of the experimental activation energy, as well as its variation, appear to be physically sound.

**Figure 6:**
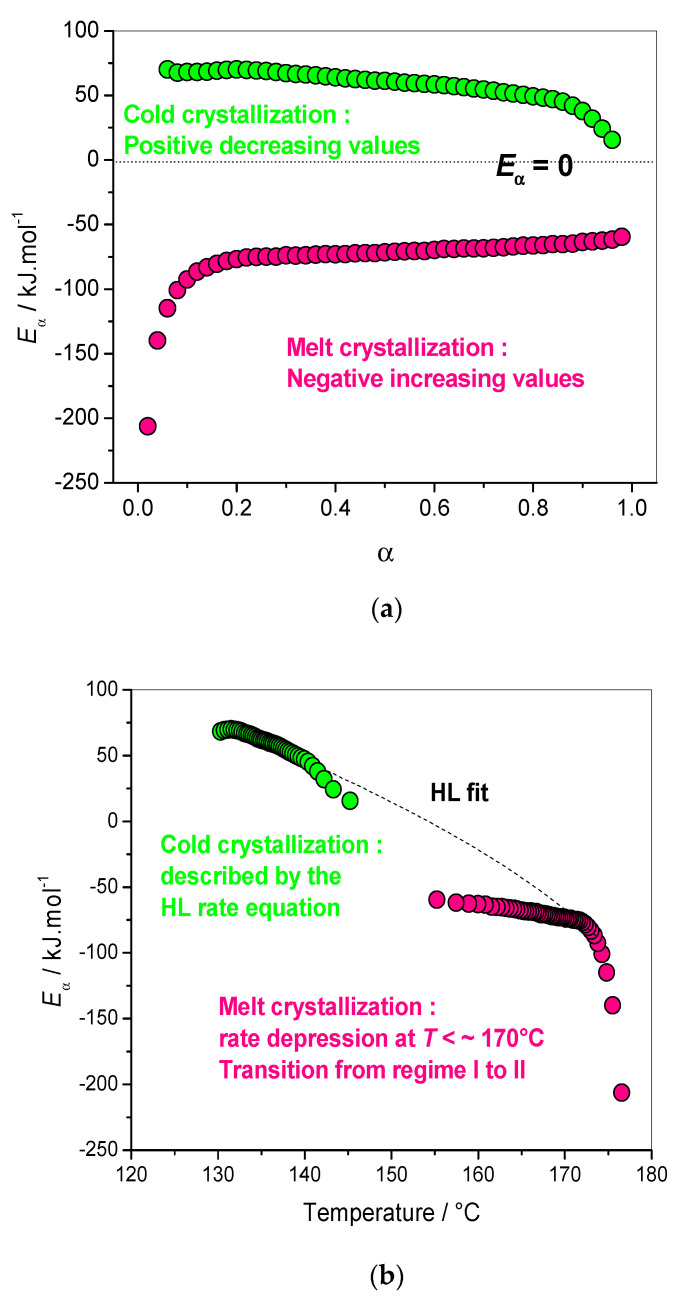
*E*_α_-dependency vs (**a**) relative degree of crystallinity (*α*), (**b**) temperature. Dash line: fit of Equation (16) for glass and melt crystallization. Adapted with permission from Copyright^©^ 2014, John Wiley and Sons [38].

The method was successfully applied to the study of the crystallization of a new and promising biobased polymer, the poly(ethylene 2,5-furandicarboxylate) (PEF). It was shown that crystallization upon heating can be described by the classical HL rate equation, while melt crystallization shows an unexpected rate depression compared to the HL predictions (Figure 6). For the melt crystallization, it was shown that nucleation governs the process, which is reflected by the negative increasing values of the apparent activation energy as computed by an advanced isoconversional method. In the cold crystallization region (crystallization from the glassy state), it was shown that diffusion governs the process and is the rate-limiting step for crystallization. Analysis of the *E_α_*-dependence has shown the existence of a change in the crystallization mechanism from the liquid at 170 °C. This result was later confirmed by other authors, i.e., Stoclet et al. and Tsanaktsis et al., using isothermal DSC, small angle X-ray diffraction (SAXS) and wide-angle X-ray Scattering (WAXS) measurements, which found that a defective crystalline form was induced upon isothermal crystallization at *T*_c_ ≤ 170 °C, while a more ordered one was induced above this critical *T*_c_ [46,47]. An atypical depression of the crystallization rate for crystallization during cooling from the melt at *T* < ~170 °C has been demonstrated, and was attributed to a change in the crystallization regime. The unexpected depression of the melt crystallization rate compared to the Hoffman–Lauritzen predictions was explained by transition to a regime where sporadic secondary nucleation limits the lateral growth rate. The faster crystallization rate, found when PEF was crystallized from the glass, suggests the more pronounced role of the other mechanisms, such as primary nucleation, at lower temperatures. The Hoffman–Lauritzen parameters *U* * and *K*_g_ were used to calculate the temperature corresponding to the maximum crystal growth rate (167 °C), which was also confirmed experimentally a year later. These parameters give information on the phenomena of nucleation and diffusion. Thus, crystallization on cooling from the melt is controlled by nucleation, and crystallization on heating (from the solid) by diffusion. The PEF crystals formed during heating or cooling exhibit similar structures (WAXD), but the crystal growth dynamics differ.

The non-isothermal Polytetrafluoroethylene (PTFE) ultrafast crystallization under tremendously fast cooling rates was achieved using fast scanning calorimetry (FSC) and ultra-fast scanning calorimetry (UFSC). The dependence of the effective activation energy (*E_α_*) on the relative extent of crystallization reveals that PTFE crystallization follows a transition from regime II to regime III at around 315–312 °C, which highlights a progressive transition from nucleation to a crystal growth-controlled process. The *E_α_*-dependencies computed from DSC, FSC and UFSC, for a wide range of cooling rates that cover a wide range of temperatures, were in perfect continuity, showing negative increasing values that tend to zero at the temperature of maximum growth rate, in agreement with the HL theory [36]. For nanocomposites based on PTFE and nanosilica, it appears from the HL parameters computed using the *E_α_*-dependencies that the nanosilica promotes the crystallization at slow cooling rates by inducing a nucleating effect, but appears to hinder the crystallization at fast cooling rates (i.e., for temperatures located further from the melting), and that the nanosilica promotes the formation of stable and more perfect crystals even when high cooling rates are employed [42].

The study of polydimethylsiloxane (PDMS) silica nanocomposites also confirmed the anti-arrhenius behavior in the melt crystallization region, and the arrhenius behavior in the glass crystallization region [37]. The study shows that glass and melt crystallizations share the same dynamics. Nucleation is promoted in the presence of silica and crystal growth is favored. Secondary crystallization is enhanced in the presence of silica nanoclusters, and higher crystal perfection is obtained. Similar conclusions were drawn for poly(butylene succinate) (PBS)/Graphene nanocomposites. The secondary crystallization upon heating is promoted with the insertion of graphene, and higher crystal perfection is obtained. A decrease of the nucleation barrier and an increase of the crystal growth rate are observed in the presence of graphene, as reflected by the values of the HL parameters U* and *K*_g_ evaluated from the *E_α_*-dependencies. This resulted in an increase of the crystal growth rate, explained by the simultaneous enhancement of nucleation and diffusion contributions in the presence of graphene nanosheets [41].

### 3.3. Relaxation and Glass Transition Kinetics

The *E_α_*-dependency is computed according to the following Equations (3) and (4). The extent of conversion during the glass transition *α* can be evaluated from DSC data with the normalized heat capacity, as follows:

(17) $$C_{p}^{N}=\frac{{\left(C_{p}-C_{pg}\right)|}_{T}}{{\left(C_{pe}-C_{pg}\right)|}_{T}}$$

where *C*_P_ is the observed heat capacity, and *C*_pg_ and *C*_pe_ are respectively the glassy and equilibrium (liquid) heat capacity. Because the values of *C*_pg_ and *C*_pe_ are temperature-dependent, they must be extrapolated into the glass transition region. Then, an advanced isoconversional method is applied. Analysis of the *E_α_*-dependence has shown a decrease in the activation energy of the relaxation, which reflects an increase in the molecular mobility when the system changes from the glassy state to the rubbery state upon heating [48]. The application of isoconversional analysis to the glass transition in various glassy systems has shown that the variation in the activation energy correlates with the dynamic fragility of the glass-forming liquids, being especially great for polymers [49]. This decrease is in agreement with the cooperative character of this transition, and has been found for several different polymers [37,48,49,50].

**Figure 7:**
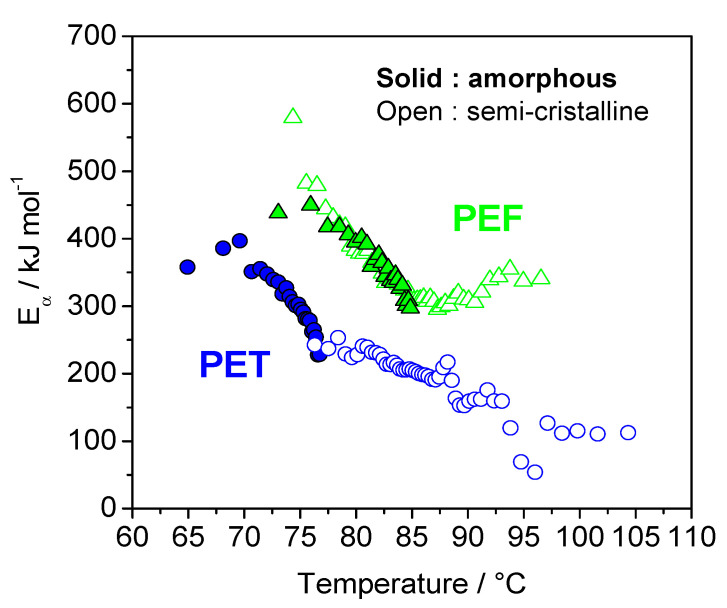
Dependence of the effective activation energy *E*_α_ upon temperature, for amorphous PEF (solid triangle), semi-crystalline PEF (open triangle) (~31% crystallinity), amorphous PET (solid circle) and semi-crystalline PET (open circles) (~34% crystallinity). Adapted and reproduced from with permission from the Royal Society of Chemistry [50].

The variation in the effective activation energy (*E_α_*) during the glass transition, for both poly(ethylene terephthalate) (PET) and PEF amorphous and semi-crystalline samples, as a function of temperature, exhibits a decrease in the effective activation energy for all samples (Figure 7). This decrease can be interpreted in terms of the cooperative motion of the chain segments involved in the glass transition. In the glassy state, only local motions of the chain segments may occur because of the very low molecular mobility. As the temperature rises, the molecular motion increases, and the translational motion of the segments and the whole chain becomes possible. This results in a decrease of the energetic constraints, which is reflected in a decrease of the effective activation energy. The effective activation energies (*E_α_*) are lower for PET, compared to the values obtained for PEF. The values obtained for amorphous PET are higher and correspond to a lower temperature domain, as compared with the values of the semi-crystalline samples. In addition, these values are in perfect continuity with the values obtained for amorphous samples. The situation is very different for PEF. The values are very close for both amorphous and semi-crystalline PEF. These results confirm the hypothesis of a reduced coupling between amorphous and crystal phases for PEF, in agreement with results obtained from the evaluation of *T*_g_ for the Mobile Amorphous Fraction (MAF) of PET, and of PET by TMDSC and DMA. Thus, analysis of the *E_α_*-dependencies has confirmed the conclusion that the relaxation of MAF is less influenced by the presence of crystals in PEF than in PET [50].

## 4. Conclusions

Advanced isoconversional methods can treat isothermal and nonisothermal data, as well as data provided from different techniques (i.e., DSC, rheometry, DMA), using the same equation. This is very attractive with regard to getting complementary information, especially in the case of complex reactions that are very dependent on the temperature domain investigated and that often need to be analyzed from the liquid state (DSC, calorimetry, FTIR, rheometry) to the solid state (TGA, DMA). This is very informative because some techniques are more sensitive at the beginning of the reaction, when the material is liquid, and less sensitive at the end when the material is highly viscous or solid, as is the case in polymerization. In this case, rheometry is a very complementary tool to DSC for extracting kinetic information on the viscous system, and DMA allows the obtaining of kinetic parameters in the solid state, when the material is cured but reactions are still in progress. In addition, FTIR and NMR data give additional information at the molecular level, used to confirm the hypotheses drawn regarding the proposed mechanism. The use of Fast Scanning Calorimetry (FSC) permits one to considerably enlarge the temperature domain for the computations. The examples presented in this study show that physically sounded kinetic information can be obtained from isoconversional kinetic analysis, that the parameters obtained can be used as starting values for nonlinear model-fitting methods, and that the exploitation of the complementarity of experimental technics and kinetic methods can be very valuable for the elucidation of complex reaction mechanisms.
